# Psychiatric Diagnoses and Risk of Suicidal Behaviour in Young Disability Pensioners: Prospective Cohort Studies of All 19-23 Year Olds in Sweden in 1995, 2000, and 2005, Respectively

**DOI:** 10.1371/journal.pone.0111618

**Published:** 2014-11-03

**Authors:** Ulf Jonsson, Kristina Alexanderson, Linnea Kjeldgård, Ellenor Mittendorfer-Rutz

**Affiliations:** Division of Insurance Medicine, Department of Clinical Neuroscience, Karolinska Institutet, Stockholm, Sweden; Peking University, China

## Abstract

**Objective:**

Increasing rates of disability pension (DP) have been observed among young adults. We studied specific psychiatric DP diagnoses and subsequent risk of suicidal behaviour in a series of three cohorts of young adult in Sweden.

**Method:**

In a nationwide register study, we included all young adults who in 1995, 2000, and 2005, respectively, were 19–23 years old and lived in Sweden (n≈500,000 per cohort). Rates of DP and specific psychiatric DP diagnoses were recorded in each cohort. Hazard ratios (HRs) and 95% confidence intervals (CIs) for suicidal behaviour during the following five years, with the corresponding age group as reference, were calculated by Cox proportional hazard regression, adjusted for demographic variables and previous own and parental suicidal behaviour.

**Results:**

The overall proportion with DP in this age group increased from 0.92% in 1995 to 2.29% in 2005, with particularly large increases in psychiatric diagnoses such as hyperkinetic disorders, pervasive developmental disorders, and depression/anxiety. The overall proportion of young disability pensioners attempting suicide during the five-year follow-up increased from 2.21% in the 1995 cohort to 3.81% in the 2005 cohort. Within most psychiatric DP diagnoses, the risk of attempted suicide did not change significantly over time, whereas suicide attempts increased in the reference group. Accordingly, the HRs for suicide attempt decreased in some psychiatric DP diagnoses. The highest adjusted HRs were observed for depression/anxiety (16.41; CI: 9.06 to 29.74) and schizophrenia (9.37; 6.13 to 14.31) in the 1995 cohort. The rate of suicide among young disability pensioners during follow-up ranged from 0.19% in 1995 to 0.37% in 2005, mainly occurring in individuals with psychiatric diagnoses.

**Conclusion:**

Suicidal behaviour has become more prevalent among young disability pensioners, which co-occurred with an increased tendency to grant DP in psychiatric diagnoses with a known high risk of suicidal behaviour. Preventive measures are warranted.

## Introduction

Disability pension (DP) in young adults has increased dramatically in Sweden and other countries within the Organisation for Economic Co-operation and Development (OECD) over the last decades, particularly due to psychiatric diagnoses. [1], [2], [3] DP is intended to provide financial security for individuals with impaired work capacity due to disease or injury. [4] However, the effects could be double-edged if the young adults granted DP are exposed to social isolation, inactivity and unhealthy living. It is therefore important to increase the knowledge about this expanding group of young adults and their risk of adverse mental health outcomes.

The increasing incidence of DP in this age group could have multiple explanations. First, changes in social insurance policies could be an important factor. [3], [5] In 2003, policies in Sweden for granting DP for young adults below age 30 years changed considerably. From that year onwards, DP can temporarily be granted to young adults with impaired work capacity or delayed completion of upper secondary school due to disease or injury. [4] Before 2003, disability pension could be granted to young individuals, both temporarily and permanently, for impaired work capacity.

There might also be other factors contributing to the specific increase in DP with psychiatric diagnoses. Changes to the definitions of mental disorders and an increased awareness of these conditions have coincided with the increase of DP with these diagnoses. Notably, both the American Psychiatric Association and the World Health Organization published updated versions of their diagnostic manuals in the early 1990’s, [6], [7] introducing changes to the diagnostic criteria for several mental disorders and adding some new disorders (e.g., Asperger syndrome). Also the growing awareness of the impairment in role functioning associated with mental disorders [8], [9], [10] could have influenced the likelihood of being granted DP in these diagnoses.

Simultaneously, psychiatric inpatient care and suicide attempt have become more prevalent in the age group 15–24 years while opposite patterns are observed for other age groups. [11] This suggests that there is also a possibility that mental health problems have become more prevalent in this specific age group. In order to better understand the mechanisms behind the increasing rates of DP in psychiatric diagnoses, it would be of great value to clarify if the increase is particularly marked for some specific diagnoses, and to shed light on if psychiatric care and socio-demographic characteristics in this group of young adults have changed over time.

There is also reason to be concerned about the risk of severe adverse mental health outcomes among young disability pensioners, and how this risk has changed as the group expanded. Suicide attempt can be viewed as the outmost consequence of mental ill-health and represents a considerable public health problem in young people. [12] We recently showed that having DP in young adulthood due to both somatic diagnoses and a range of psychiatric diagnoses was associated with an increased risk of suicide attempt, beyond the effects of the individual’s socio-economic status, previous suicide attempts and parental risk factors. [13] The study also revealed that half of the young people with a psychiatric DP diagnosis who attempted suicide during a five-year follow-up previously had been treated for suicide attempts, [13] indicating a continuing course of this severe problem. It is not known how suicidal behaviour has changed over time among young adults on DP.

### Aim

The aims were to investigate psychiatric DP diagnoses among young adults over time, and to scrutinise the risk of suicidal behaviour in this specific group of young adults.

## Methods

**Figure 1:**
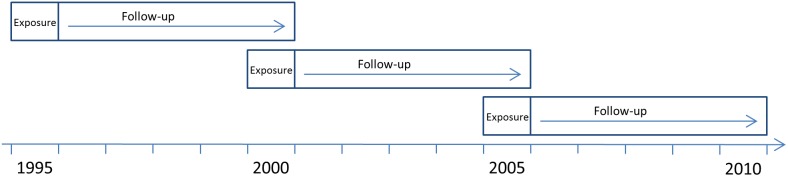
Timeline of the three cohorts. The three cohorts included all individuals 19–23 years of age and living in Sweden in 1995, 2000, and 2005, respectively. Each cohort was followed for five years from the year after inclusion.

Three prospective population-based cohort studies were conducted using linked de-identified register data from several nationwide Swedish registers. The cohorts included all individuals who were 19–23 years old and lived in Sweden all of the year 1995, 2000, or 2005, respectively. Each cohort was followed up regarding suicide attempt and suicide for five years after the year they were included in respective cohorts (1996–2000; 2001–2005, and 2006–2010) (Figure 1).

### Ethics

The study population was based on linkage of several public national registers. Ethical vetting is always required when using register data in Sweden. The ethical vetting is performed by regional ethical review boards and the risk appraisal associated with the Law on Public Disclosure and Secrecy is done by data owners. The ethical review boards can however waive the requirement to consult the data subjects (or in case of minors/children the next of kin, careers or guardians) directly to obtain their informed consent, and will often do so if the research is supported by the ethical review board and the data has already been collected in some other context. This means that for this specific study no written informed consent was given by participants (or next of kin/caregiver in the case of children) for their clinical records to be used. Patient records/information was anonymized and de-identified prior to analysis. This was done by Statistics Sweden, the authority responsible for data linkage. Researchers received de-identified data. This project has been evaluated and approved by the Regional Ethical Review Board of Karolinska Institutet, Stockholm, Sweden (protocol nr 2007/762-31).

### DP diagnoses

Data on the main DP diagnosis was obtained from the National Social Insurance Agency. The diagnoses were classified according to the International Statistical Classification of Diseases and Related Health Problems 10^th^ Revision (ICD-10) [6] and categorized into psychiatric diagnoses (F00–F99) and somatic diagnoses (all other diagnostic codes), and missing information, respectively.

The psychiatric DP diagnoses were categorized into the following subgroups: “Schizophrenia” including schizophrenia, schizotypal and delusional disorders (F20–F29); “Depression/anxiety” including unipolar depression/neurotic, stress-related and somatoform disorders (F32–F33, F40–F49); “Mental retardation” (F70–F79); “Pervasive developmental disorder” (F84); “Hyperkinetic disorder” (F90); and “Other mental disorders” (F00–F19, F30–F31, F34–F39, F50–F59, F60–F69, F80–F83, F85–F99).

### Socio-demographic variables

Information about sex, country of birth, and parental education was obtained from the Longitudinal Integrated Population-based Database for Labour-market Research (LISA) held by Statistic Sweden, and then linked with the Multi-Generational Register. Country of birth was classified as either Sweden or other. Parental level of education registered five years before inclusion in the cohort (1990, 1995, and 2000 respectively) was classified as low (compulsory school or less, ≤9 years), medium (upper secondary school, 10–12 years), or high (college or university, >12 years). We used either maternal or paternal education, whichever was the highest, in accordance with the dominance principle [14].

### Suicidal behaviour and psychiatric inpatient care

Information on suicide attempts and suicides among the participants was obtained from the National Patient Register and the Cause of Death Register, held by the National Board of Health and Welfare. Suicide attempts and completed suicides were classified according to the ICD-10, [7] defined by the codes X60–X84 and Y10–Y34. Codes with uncertainty about intention (Y10–Y34) were included, primarily in order to limit temporal and regional variation in ascertainment routines. [15], [16] Suicide attempt was measured in two separate periods for each cohort: for the five years following the year the cohort was defined (e.g., from 1 January 2006 to 31 December 2010 for the cohort of 2005), and anytime before the baseline.

Information about previous inpatient care with a psychiatric diagnosis was retrieved for the 6 years preceding baseline (e.g., from 1 January 2000 to 31 December 2005 for the cohort of 2005). Psychiatric diagnosis was defined as any mental and behavioural disorder in ICD-10 (F00–F99).

### Parental suicidal behaviour

Information on parental suicide attempts between 1964 and 31 December of the inclusion year (1995, 2000 and 2005 respectively) was obtained from the National Patient Register and defined by the ICD-9 codes E950–E959 and E980–E989 or the ICD-10 codes X60–X84 and Y10–Y34. Information on parental suicide from the year when the child was born to 31 December the year the cohort was defined was obtained from the Causes of Death Register. Events of parental suicide attempts or suicide were combined to one dichotomized measure of suicidal behaviour.

### Statistical analyses

Differences between the cohorts regarding DP diagnoses, socio-demographic variables, inpatient care and suicidal behaviour were tested with the χ^2^-test. We calculated the Hazard Ratios (HR) with 95% confidence intervals (CI) for suicide attempt and suicide during the five years following inclusion in the cohort. Cox proportional hazard regression models were used, with person-time during follow-up as the underlying time scale. The data conformed to the proportional hazards assumption. Individuals were followed from 1 January the year following inclusion in the cohort to the event, death, emigration, and end of follow-up, whichever came first. We performed both univariate and multivariate analyses. In a first set of analyses, the categories of psychiatric DP diagnoses, DP with somatic diagnoses, and missing information on DP diagnoses were contrasted to no DP (reference category). In a second set of analyses, DP due to all the subgroups of psychiatric diagnoses and the category of other diagnoses were contrasted to no DP (reference category). In the analyses of suicide, division into different diagnostic subgroups was not possible due to lack of power. The statistical analyses were performed with IBM SPSS 20.

## Results

The study populations consisted of 559,147 individuals in the cohort of 1995, 504,741 individuals in the cohort of 2000, and 525,276 individuals in the cohort of 2005. The proportion with DP gradually increased: 0.92% in the 1995 cohort, 1.17% in the 2000 cohort, and 2.29% in the 2005 cohort from 2005 (Table 1).

**Table 1:** Demographic data for young adults 19–23 years old with and without disability pension (DP) in the three studied cohorts of 1995, 2000, and 2005, respectively.

		All in the cohort	Men	Low parental education (≤9 years)	Country of birth: not Sweden
DP diagnosis	Cohort	n (%)	n (%)	n (%)	n (%)
No DP	1995	553 989 (99.08)^ab^	282 949 (51.07)	118 284 (21.35)^ab^	48 358 (8.73)^ab^
	2000	498 827 (98.83)^ac^	254 793 (51.08)	83 758 (16.79)^ac^	51 005 (10.22)^ac^
	2005	513 231 (97.71)^bc^	263 071 (51.26)	68 602 (13.37)^bc^	63 128 (12.30)^bc^
All DP	1995	5 158 (0.92)^ab^	2 817 (54.61)	1 416 (27.45)^ab^	387 (7.50)^ab^
	2000	5 914 (1.17)^ac^	3 260 (55.12)	1 426 (24.11)^ac^	649 (10.97)^ac^
	2005	12 045 (2.29)^bc^	6 558 (54.45)	2 320 (19.26)^bc^	1 500 (12.45)^bc^
					
Psychiatric DP	1995	1 767 (0.32)^ab^	942 (53.31)	550 (31.13)^ab^	155 (8.77)^ab^
	2000	3 127 (0.62)^ac^	1 744 (55.77)	844 (26.99)^ac^	348 (11.13)^a^
	2005	8 070 (1.54)^bc^	4 441 (55.03)	1 623 (20.11)^bc^	977 (12.11)^b^
Somatic DP	1995	1 280 (0.23)^ab^	645 (50.39)	295 (23.05)^b^	90 (7.03)^ab^
	2000	1 937 (0.38)^ac^	1 023 (52.81)	403 (20.81)^c^	224 (11.56)^ac^
	2005	3 419 (0.65)^bc^	1 784 (52.18)	614 (17.96)^bc^	777 (11.71)^bc^
					
DP diagnosis	1995	2 111 (0.38)^ab^	1 230 (58.27)	571 (27.05)^ab^	142 (6.73)^ab^
missing	2000	850 (0.17)^ac^	493 (58.00)	179 (21.06)^ac^	77 (9.06)^a^
	2005	556 (0.11)^bc^	333 (59.89)	83 (14.93)^bc^	60 (10.79)^b^

a Statistical difference (p<0.05) between 1995 and 2000.

b Statistical difference (p<0.05) between 1995 and 2005.

c Statistical difference (p<0.05) between 2000 and 2005.

### Diagnoses for which DP was awarded

**Figure 2:**
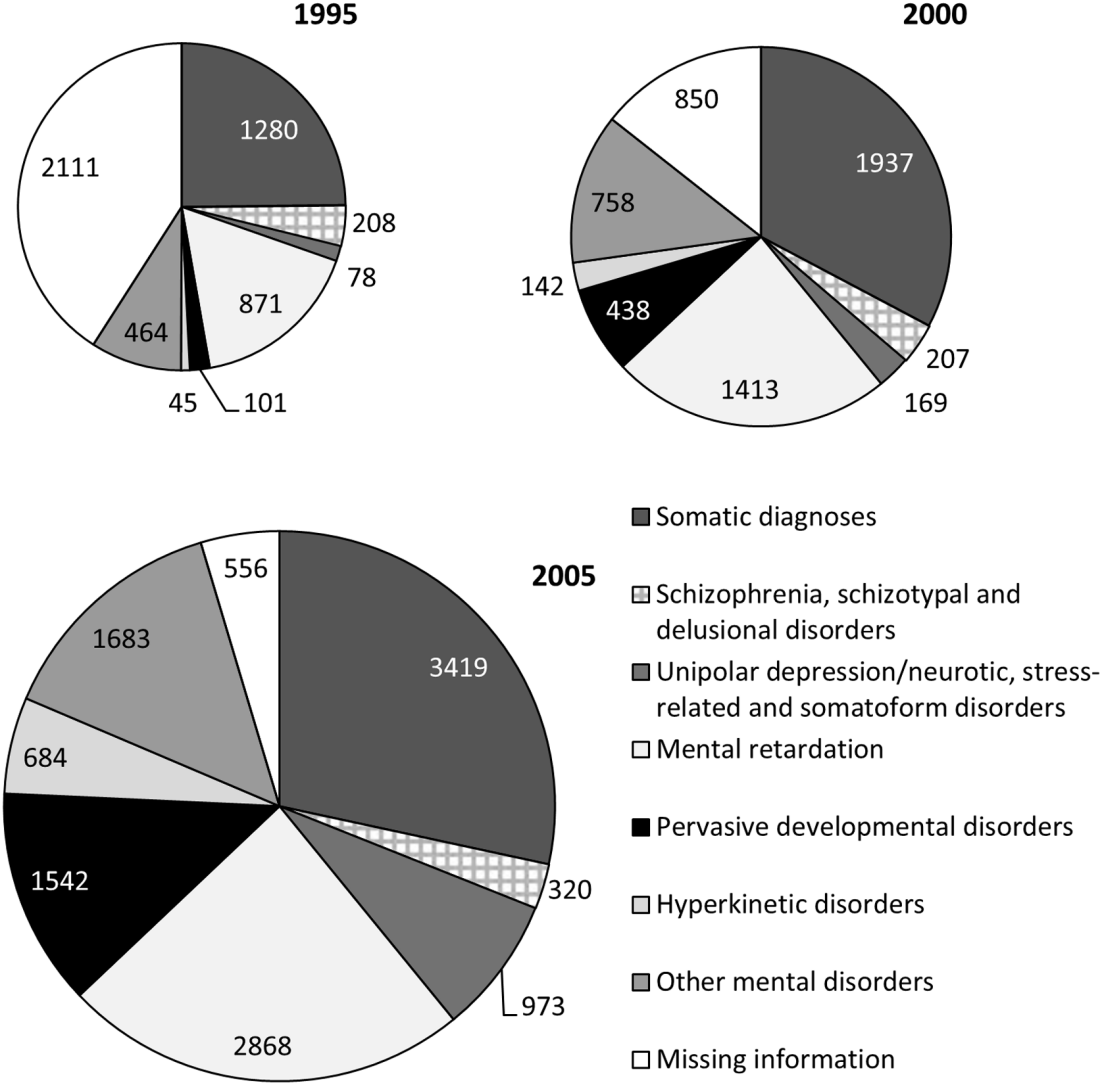
The diagnoses for which disability pension was granted within each cohort. The number of young adults aged 19–23 years on disability pension in the years 1995, 2000 and 2005, divided into diagnostic groups. The relative size of the diagrams represents the proportion of young adults in the specific age group granted disability pension.

The gradual increase in the number of young adults with DP occurred largely in psychiatric diagnoses, increasing fivefold in a period of only 10 years, from 0.32% in 1995 to 1.54% in 2005 (Table 1). The proportion with DP gradually increased over time in all six specific psychiatric diagnostic groups (Table 2). There was a particularly large increase within the diagnostic subgroups of hyperkinetic disorder, pervasive developmental disorder, and depression/anxiety (Figure 2).

**Table 2:** Demographic data for young adults 19–23 years old with disability pension (DP) due to specific psychiatric diagnoses in the three studied cohorts of 1995, 2000, and 2005, respectively.

		All in the cohort	Men	Low parental education(≤9 years)	Country of birth: not Sweden
DP diagnosis	Cohort	n (%)	n (%)	n (%)	n (%)
Schizophrenia	1995	208 (0.04)^b^	126 (60.58)	50 (24.04)	30 (14.42)
	2000	207 (0.04)^c^	130 (62.80)	53 (25.60)^c^	29 (14.01)
	2005	320 (0.06)^bc^	199 (62.19)	55 (17.19)^c^	61 (19.06)
Depression/anxiety	1995	78 (0.01)^ab^	36 (46.15)	23 (29.49)^b^	6 (7.69)
	2000	169 (0.03)^ac^	72 (42.60)	39 (23.08)	11 (6.51)
	2005	973 (0.19)^bc^	360 (37.00)	166 (17.06)^b^	101 (10.38)
Mentalretardation	1995	871 (0.16)^ab^	452 (51.89)	301 (34.56)^b^	75 (8.61)^ab^
	2000	1 413 (0.28)^ac^	744 (52.65)	450 (31.85)^c^	176 (12.46)^ac^
	2005	2 868 (0.55)^bc^	1 577 (54.99)	767 (26.74)^bc^	431 (15.03)^bc^
Pervasive developmental disorder	1995	101 (0.02)^ab^	77 (76.24)	21 (20.79)^b^	6 (5.94)
	2000	438 (0.09)^ac^	305 (69.63)	82 (18.72)^c^	40 (9.13)
	2005	1 542 (0.29)^bc^	1 067 (69.20)	186 (12.06)^bc^	134 (8.69)
Hyperkinetic	1995	45 (0.01)^ab^	32 (71.11)	8 (17.78)	5 (11.11)
disorder	2000	142 (0.03)^ac^	105 (73.94)	35 (24.65)^c^	13 (9.15)
	2005	684 (0.13)^bc^	488 (71.35)	115 (16.81)^c^	63 (9.21)
Otherpsychiatric	1995	464 (0.08)^ab^	219 (47.20)	147 (31.68)^ab^	33 (7.11)^b^
	2000	758 (0.15)^ac^	388 (51.19)^c^	185 (24.41)^ac^	79 (10.42)
	2005	1 683 (0.32)^bc^	750 (44.56)^c^	334 (19.85)^bc^	187 (11.11)^b^

a Statistical difference (p<0.05) between 1995 and 2000.

b Statistical difference (p<0.05) between 1995 and 2005.

c Statistical difference (p<0.05) between 2000 and 2005.

### Socio-demographic and parental characteristics

In all three cohorts, there was an overrepresentation of men among those with psychiatric DP diagnosis (53 to 56% men). The majority of the young adults with DP due to schizophrenia, pervasive developmental disorders, and hyperkinetic disorder were men, while the majority with DP due to depression/anxiety were women (Table 2). The male/female ratio within the diagnostic subgroups did not differ significantly between the cohorts, with the exception of the category of other psychiatric diagnoses (Table 2). There was an increase in parental educational level both amongst those not on DP and those on DP with psychiatric diagnoses. There was also a similar gradual increase of individuals born outside Sweden among both those without DP and those on DP with psychiatric diagnoses. Finally, there was a slight gradual increase in the likelihood that a parent had committed suicide or had been treated for suicidal behaviour both among those without DP and among those on DP with psychiatric diagnoses (Table 3). While 4–5% of the young adults without DP had parents with previous suicidal behaviour, the rate was 8–10% among the young adults on DP with psychiatric diagnoses.

**Table 3:** Parental suicidal behaviour, own inpatient care due to psychiatric diagnoses, suicide attempt six years prior to baseline, and inpatient care due to suicide attempt during a five-year follow up in young adults (19–23 year) with and without disability pension (DP) in 1995, 2000 and 2005, respectively.

		Parental suicidal behaviour	Previous psychiatric inpatient care	Previous suicide attempt	Suicide attempt during follow-up
DP diagnosis	Cohort	n (%)	n (%)	n (%)	n (%)
No DP	1995	22 454 (4.05)^ab^	8 342 (1.51)^ab^	5 611 (1.01)^ab^	2449 (0.44)^ab^
	2000	21 664 (4.34)^ac^	10 433 (2.09)^ac^	5 452 (1.09)^ac^	3824 (0.77)^ac^
	2005	24 644 (4.80)^bc^	12 387 (2.41)^bc^	6 474 (1.26)^bc^	4449 (0.87)^bc^
					
All DP	1995	320 (6.20)^b^	858 (16.63)	192 (3.72)^b^	114 (2.21)^b^
	2000	407 (6.88)^c^	950 (16.06)	237 (4.01)^c^	141 (2.38)^b^
	2005	1 038 (8.62)^bc^	2 004 (16.64)	761 (6.32)^bc^	459 (3.81)^bc^
Psychiatric DP	1995	133 (7.53)^b^	565 (31.98)^ab^	115 (6.51)^b^	80 (4.53)
	2000	260 (8.31)^c^	780 (24.94)^ac^	189 (6.04)^c^	107 (3.42)^c^
	2005	775 (9.60)^bc^	1 780 (22.06)^bc^	664 (8.23)^bc^	389 (4.82)^c^
Somatic DP	1995	67 (5.23)	88 (6.88)	36 (2.81)	16 (1.25)
	2000	116 (5.99)	125 (6.45)	39 (2.01)	29 (1.5)
	2005	230 (6.73)	194 (5.67)	89 (2.6)	68 (1.99)
Missing DP	1995	120 (5.68)^a^	205 (9.71)^ab^	41 (1.94)	18 (0.85)
diagnosis	2000	31 (3.65)^ac^	45 (5.29)^a^	9 (1.06)	5 (0.59)
	2005	33 (5.94)^c^	30 (5.40)^b^	8 (1.44)	<5 (0.36)

a Statistically significant difference (p<0.05) between 1995 and 2000 cohorts.

b Statistically significant difference (p<0.05) between 1995 and 2005 cohorts.

c Statistically significant difference (p<0.05) between 2000 and 2005 cohorts.

### Previous inpatient care and suicide attempts

The proportions of individuals with inpatient care due to psychiatric disorders during the six years preceding baseline and any previous suicide attempt for the different cohorts are shown in Table 3–4. While proportions with previous inpatient care due to psychiatric disorders gradually increased from the cohort 1995 to 2005 in individuals without DP, the opposite was observed for young adults with DP in psychiatric diagnoses. In the 1995 cohort, 1.51% of individuals without DP had had previous psychiatric inpatient care. The corresponding figure was 2.41% in the cohort from 2005 (Table 3). In contrast, 32% of individuals with DP due to psychiatric diagnoses received such care in the 1995 cohort. This proportion decreased significantly to 22% in 2005. A similar pattern emerged for the majority of the six specific psychiatric DP diagnoses, with one exception: DP due to hyperkinetic disorders (Table 4). The likelihood of having been treated in psychiatric inpatient care varied widely among the six specific psychiatric diagnostic groups; while around 90% of young disability pensioners with schizophrenia had received such care, the corresponding figures for mental retardations was 8–11% (Table 4).

**Table 4:** Parental suicidal behaviour, own inpatient care due to psychiatric diagnoses, suicide attempt six years prior to baseline, and inpatient care due to suicide attempt during a five-year follow-up in young adults (19–23 year) with disability pension (DP) due to specific psychiatric diagnoses in 1995, 2000 and 2005, respectively.

		Parental suicidal behaviour	Previous psychiatric inpatient care	Previous suicide attempt	Suicide attempt during follow-up
DP diagnosis	Cohort	n (%)	n (%)	n (%)	n (%)
Schizophrenia	1995	19 (9.13)	191 (91.83)^b^	32 (15.38)	22 (10.58)^a^
	2000	12 (5.80)	179 (86.47)	27 (13.04)	9 (4.35)^ac^
	2005	31 (9.69)	266 (83.13)^b^	48 (15.00)	29 (9.06)^c^
Depression/anxiety	1995	8 (10.26)	39 (50.00)^b^	11 (14.10)	11 (14.1)
	2000	18 (10.65)	76 (44.97)	23 (13.61)	15 (8.88)
	2005	107 (11.00)	370 (38.03)^b^	187 (19.22)	87 (8.94)
Mentalretardation	1995	57 (6.54)^b^	92 (10.56)^b^	5 (0.57)^ab^	5 (0.57)^b^
	2000	118 (8.35)	118 (8.35)	22 (1.56)^a^	8 (0.57)^c^
	2005	252 (8.79)^b^	217 (7.57)^b^	54 (1.88)^b^	60 (2.09)^bc^
Pervasive developmentaldisorder	1995	7 (6.93)	39 (38.61)^ab^	5 (4.95)	<5 (2.97)
	2000	28 (6.39)	107 (24.43)^ac^	12 (2.74)	8 (1.83)
	2005	129 (8.37)	262 (16.99)^bc^	65 (4.22)	43 (2.79)
Hyperkinetic	1995	<5 (8.89)	9 (20.00)	<5 (2.22)	0 (0)
disorder	2000	12 (8.45)	20 (14.08)	<5 (2.82)^c^	9 (6.34)
	2005	80 (11.70)	130 (19.01)	50 (7.31)^c^	39 (5.7)
Other psychiatric	1995	38 (8.19)	195 (42.03)^b^	61 (13.15)	39 (8.41)
	2000	72 (9.50)	280 (36.94)^c^	101 (13.32)	58 (7.65)
	2005	176 (10.46)	535 (31.79)^bc^	260 (15.45)	131 (7.78)

a Statistically significant difference (p<0.05) between 1995 and 2000 cohorts.

b Statistically significant difference (p<0.05) between 1995 and 2005 cohorts.

c Statistically significant difference (p<0.05) between 2000 and 2005 cohorts.

Suicide attempt prior to baseline gradually became more likely among the young adults without DP, from the first to the last cohort. Among the young adults on DP with psychiatric diagnoses, the risk was also higher in the last cohort (2005) than in the other two cohorts (Table 3). A similar pattern existed for several of the six specific psychiatric diagnostic groups (Table 4).

### Suicide attempt during follow up

Within the six specific psychiatric diagnostic groups, inpatient care for suicide attempts during follow-up was most common among those on DP due to depression/anxiety (9–14%) and schizophrenia (4–11%) and lower in mental retardation (1–2%) and autism spectrum disorders (2–3%) (Table 4).

There was an increasing temporal trend in proportions of young individuals treated for suicide attempts during follow up among young adults not on DP. Among the young adults on DP there was a similar increasing trend (Table 3). However, such an increase was not observed within the specific diagnostic groups. Only for mental retardation there was a significant increase from the two earlier cohorts to the last cohort (Table 4).

In all cohorts, young adults with DP due to psychiatric diagnoses had elevated crude HRs for suicide attempts during the follow-up, compared with those without DP (Table 5). For DP due to schizophrenia, depression/anxiety and the category of other psychiatric diagnoses, the unadjusted HRs of suicide attempts were lower for the 2000 and 2005 cohorts than for the 1995 cohort, while opposite time trends were seen for DP due to mental retardation (Table 6). In all analyses, risk estimates were marginally affected by adjustment for sex, country of birth, parents' educational level, and previous suicidal behaviour among parents (Table 5 and 6). After adjustment for own previous suicide attempts, estimates were decreased for DP due to psychiatric diagnoses, particularly in the 1995 cohort. However, estimates were in general still high in the fully adjusted models.

**Table 5:** Hazard ratios (HR) and 95% confidence intervals (CI) for suicide attempt during a five-year follow-up of young adults with disability pension (DP) due to psychiatric, somatic, or missing diagnoses in 1995, 2000, and 2005, respectively^1^.

		Suicide attempt	Crude model	Model 1^a^	Model 2^b^
Cohort	DP diagnosis	n (%)	HR (95% CI)	HR (95% CI)	HR (95% CI)
1995	No DP	2 449 (0.44)	1.00	1.00	1.00
	Psychiatric	80 (4.53)	10.50 (8.40–13.11)	9.52 (7.61–11.9)	6.36 (5.08–7.96)
	Somatic	16 (1.25)	2.87 (1.75–4.69)	2.77 (1.70–4.53)	2.38 (1.45–3.88)
	Missing	18 (0.85)	1.95 (1.23–3.10)	1.94 (1.22–3.09)	1.82 (1.14–2.89)
2000	No DP	3 824 (0.77)	1.00	1.00	1.00
	Psychiatric	107 (3.42)	4.53 (3.74–5.49)	4.04 (3.33–4.9)	2.87 (2.36–3.49)
	Somatic	29 (1.50)	1.98 (1.37–2.85)	1.87 (1.30–2.70)	1.74 (1.21–2.51)
	Missing	5 (0.59)	0.77 (0.32–1.86)	0.80 (0.33–1.91)	0.83 (0.35–2.00)
2005	No DP	4 449 (0.87)	1.00	1.00	1.00
	Psychiatric	389 (4.82)	5.66 (5.11–6.28)	5.07 (4.56–5.62)	3.32 (2.98–3.69)
	Somatic	68 (1.99)	2.32 (1.82–2.95)	2.17 (1.71–2.76)	1.99 (1.56–2.52)
	Missing	<5 (0.36)	0.42 (0.10–1.66)	0.42 (0.10–1.67)	0.40 (0.10–1.60)

1 Reference group comprises all young adults without disability pension in each cohort.

a Adjusted for sex, country of birth, parental educational level, and parental suicidal behavior.

b Additionally adjusted for suicide attempt in the index subjects.

**Table 6:** Hazard ratios (HR) and 95% confidence intervals (CI) for suicide attempt during a five-year follow-up of young adults with disability pension (DP) due to specific psychiatric diagnoses in 1995, 2000 and 2005, respectively^1^.

		Suicide attempt	Crude	Model 1^a^	Model 2^b^
Cohort	DP diagnosis	n (%)	HR (95% CI)	HR (95% CI)	HR (95% CI)
1995	Schizophrenia	22 (10.58)	25.63 (16.84–39.00)	23.28 (15.29–35.44)	9.37 (6.13–14.31)
	Depression/anxiety	11 (14.10)	35.66 (19.72–64.49)	28.04 (15.50–50.72)	16.41 (9.06–29.74)
	Mental retardation	5 (0.57)	1.29 (0.54–3.11)	1.16 (0.48–2.79)	1.31 (0.55–3.15)
	Pervasive developmental	<5 (2.97)	6.86 (2.21–21.27)	8.30 (2.68–25.78)	6.83 (2.20–21.20)
	Hyperkinetic	0 (0.00)	-	-	-
	Other mental	39 (8.41)	19.95 (14.54–27.37)	17.77 (12.95–24.4)	8.03 (5.83–11.07)
2000	Schizophrenia	9 (4.35)	5.85 (3.04–11.25)	5.74 (2.98–11.04)	2.71 (1.41–5.22)
	Depression/anxiety	15 (8.88)	12.13 (7.31–20.15)	10.35 (6.23–17.19)	5.12 (3.08–8.52)
	Mental retardation	8 (0.57)	0.74 (0.37–1.47)	0.62 (0.31–1.24)	0.62 (0.31–1.24)
	Pervasive developmental	8 (1.83)	2.39 (1.19–4.78)	2.50 (1.25–5.00)	2.21 (1.11–4.43)
	Hyperkinetic	9 (6.34)	8.44 (4.39–16.24)	8.42 (4.38–16.21)	7.67 (3.99–14.75)
	Other psychiatric	58 (7.65)	10.44 (8.06–13.54)	9.18 (7.08–11.91)	4.43 (3.41–5.77)
2005	Schizophrenia	29 (9.06)	10.95 (7.60–15.77)	10.69 (7.42–15.40)	4.86 (3.37–7.02)
	Depression/anxiety	87 (8.94)	10.90 (8.81–13.47)	9.13 (7.38–11.29)	4.08 (3.29–5.07)
	Mental retardation	60 (2.09)	2.42 (1.87–3.12)	2.07 (1.60–2.67)	2.03 (1.57–2.62)
	Pervasive developmental	43 (2.79)	3.23 (2.39–4.36)	3.33 (2.47–4.50)	2.68 (1.98–3.61)
	Hyperkinetic	39 (5.70)	6.66 (4.86–9.13)	6.25 (4.56–8.57)	3.98 (2.90–5.47)
	Other psychiatric	131 (7.78)	9.32 (7.83–11.08)	7.95 (6.68–9.46)	3.93 (3.29–4.69)

1 Reference group comprises all young adults without disability pension in each cohort.

a Adjusted for sex, country of birth, parental educational level, and parental suicidal behavior.

b Additionally adjusted for suicide attempt in the index subjects.

### Suicide during follow-up

In all three cohorts, there was an elevated risk of suicide for young adults on DP due to psychiatric diagnoses (Table 7). The proportion among young adults with no DP that committed suicide within the five years of follow-up was 0.06% in the first cohort and 0.08% in the other cohorts. Among the total group of young adults with DP, the rates of suicide during follow-up changed from 0.19% in the 1995 cohort to 0.37% in the 2005 cohort. The differences between the cohorts were not statistically significant. The proportion of those with DP due to psychiatric diagnoses that committed suicide within the follow-up ranged from 0.40% in the 1995 cohort to 0.52% in the 2005 cohort. The unadjusted regression models showed HRs of around 6–7 for the group with psychiatric DP diagnoses in all three cohorts. Adjustment due to the different covariates decreased the estimates somewhat and multivariate HRs for DP due to psychiatric diagnoses ranged between 3.9 and 4.3 in the cohorts. Among the young adults on DP, suicide occurred mainly in individuals with DP in psychiatric diagnoses.

**Table 7:** Hazard ratios (HR) and 95% confidence intervals (CI) for suicide during a five-year follow-up of young adults with disability pension (DP) due to psychiatric**,** somatic, or missing diagnoses in 1995, 2000, and 2005, respectively^1^.

		Suicide	Crude	Model 1^a^	Model 2^b^
Cohort	DP diagnosis	n (%)	HR (95% CI)	HR (95% CI)	HR (95% CI)
1995	No DP	314 (0.06)	1.00	1.00	1.00
	Psychiatric	7 (0.40)	6.96 (3.29–14.71)	6.20 (2.93–13.14)	4.26 (2.00–9.09)
	Somatic	<5 (0.16)	2.79 (0.70–11.22)	2.75 (0.69–11.06)	2.32 (0.58–9.31)
	Missing	<5 (0.05)	0.85 (0.12–6.03)	0.77 (0.11–5.47)	0.71 (0.10–5.08)
2000	No DP	395 (0.08)	1.00	1.00	1.00
	Psychiatric	14 (0.45)	5.65 (3.32–9.63)	4.95 (2.90–8.44)	3.52 (2.04–6.05)
	Somatic	<5 (0.21)	2.64 (0.98–7.06)	2.45 (0.91–6.56)	2.39 (0.89–6.40)
	Missing	<5 (0.12)	1.50 (0.21–10.67)	1.46 (0.20–10.37)	1.46 (0.20–10.37)
2005	No DP	404 (0.08)	1.00	1.00	1.00
	Psychiatric	42 (0.52)	6.74 (4.92–9.23)	5.8 (4.22–7.96)	3.92 (2.83–5.44)
	Somatic	<5 (0.06)	0.75 (0.19–3.00)	0.7 (0.18–2.83)	0.63 (0.16–2.52)
	Missing	0 (0)	-	-	-

1 Reference group comprises all young adults without disability pension in each cohort.

a Adjusted for sex, country of birth, parental educational level, and parental suicidal behavior.

b Additionally adjusted for suicide attempt in the index subjects.

## Discussion

This is the first prospective study of suicidal behaviour among young disability pensioners in a series of cohorts of young adults. The number of young adults on DP increased sharply over the period from 1995 to 2005, particularly due to psychiatric diagnoses such as hyperkinetic disorder, pervasive developmental disorder, and depression/anxiety. Parallel to these trends, the proportion of young disability pensioners with subsequent suicidal behaviour increased. For most diagnostic subgroups no increase was observed, indicating that the increase among young disability pensioners in general was driven by the large expansion of diagnostic groups with a documented high risk of suicidal behaviour.

The increase of DP in psychiatric diagnoses is in line with recent findings from several countries. [17] Our results seem to reflect the dramatic rise from the mid 1990’s in diagnoses such as hyperkinetic disorder (or attention-deficit hyperactivity disorder) and pervasive developmental disorder in the USA and Europe. [18], [19], [20], [21] The increase of young people on DP with these diagnoses might partly be attributable to changes in the early 1990s of the diagnostic systems of the American Psychiatric Association [7] and the World Health Organization. [6] There has also been an increasing awareness of the impairment in role functioning associated with these and other mental disorders, [8], [9], [10] which is likely to have had an impact on the likelihood of being granted DP in these diagnoses.

The increasing rates of DP with psychiatric diagnoses underscores that reliable and valid measures of functional impairment related to mental disorders are required, in order to secure equal assessments over time and between practitioners. Of note, the proportion with previous psychiatric inpatient care among the individuals with psychiatric DP diagnoses decreased from the 1995 to the 2005 cohort. In individuals of the same age without DP, this pattern was inverted, with a gradual increase in the proportion of individuals who received such care. Thus, it seems plausible that increasing knowledge about psychiatric diagnoses and the disability associated with such conditions has resulted in that young individuals with less severe symptoms (i.e., not requiring psychiatric inpatient care) are granted DP. However, it is equally plausible that the criteria for granting DP with psychiatric diagnoses gradually have become more lenient. Reliable standards for assessing functional impairment in patients with mental disorders have not yet been established. The ongoing efforts to categorize aspects of functioning that are likely to be affected in specific mental disorders, according to the International Classification of Functioning, Disability, and Health (ICF), [8], [9] could prove to be a major step forward in establishing such standards.

If we presume that the increase in DP with psychiatric diagnoses in part is attributable to an increased tendency to grant DP for less severe forms of the disorders, a decrease in severe outcomes such as suicidal behaviour among these young adults could be expected. A decrease in severe outcomes might also be expected, given the substantial change in clinical practice and availability of psychiatric care since 1990’s, with for instance a steady increase in prescription of antidepressants. [22] Still, the risk of suicidal behaviour remained high over time among young adults on psychiatric DP. The relative risk of suicide attempt among young adults on DP due to psychiatric diagnoses decreased over time (compared with all other young adults of the same age not on DP). However, this was mainly explained by a steady increase in suicide attempts from 1995 to 2005 among the age group as a whole (the reference group) and not by a decrease among those on DP.

The continued high risk of suicidal behaviour in this expanding group is disconcerting. A recent study showed that the rate of suicide attempts among young adults with DP increased continuously up to the year preceding the granting of DP, after which it declined. [23]. However, the present study shows that there still is a high risk among those who have been granted DP. There was a clear attenuation of the risk estimates of subsequent suicide attempt in all cohorts after adjustment for previous suicide attempt. This suggests that a significant proportion of those with DP due to psychiatric diagnoses with suicide attempts during follow-up also had been treated for suicide attempt previously, highlighting the need for suicide risk assessments and adequate interventions in this high risk group [24], [25], [26].

### Strengths and limitations

Strengths of this study include the prospective, population-based cohort design, the use of nationwide register data of high quality, [27] and that also information on the parents of the young adults could be included. In addition, a number of confounding factors have been taken into account. Another strength is the large size of the three cohorts, allowing analyses of sub groups, including different diagnostic categories and relatively rare outcomes like suicide.

Some limitations should be noted. First, societal changes and other external events (e.g., changes in social insurance policies, educational systems, availability of health care, and migration) will inevitably diminish the comparability of the cohorts. The comparability is further compromised by any changes in diagnostic procedures occurring over the time separating the first and the last cohort.

Further, outpatient visits due to these diagnoses were not included, which means that we mainly had information about the more severe disorders and attempts. A study conducted in Sweden estimated that only up to half of suicide attempters are treated. [28] The information about the number of suicide attempts presented in this report is, therefore, rather an underestimation than an overestimation, and it is not clear whether underreporting differed over time. Further, the validity of the DP diagnoses is uncertain. To the best of our knowledge, there is no study on the validity of DP diagnoses. However, one study exists on the validity of sick-leave diagnoses, showing high validity when comparing to diagnoses from medical records [29].

Another limitation is that the register from the Social Insurance Agency did not include information on diagnosis for a significant proportion of disability pension in the 1995 cohort. Therefore, we have consistently chosen to report results separately for the group for which DP diagnoses were missing. The data shows that this group resembled more closely the group with somatic diagnoses and mental retardation than any other psychiatric diagnostic groups. We, therefore, find it unlikely that the increase observed in hyperkinetic disorder, pervasive developmental disorder, and depression/anxiety would have been attenuated much if information about all DP diagnoses had been available.

### Conclusions

Parallel to the large expansion of the group of young adults receiving DP in psychiatric diagnoses, suicide attempt and completed suicide have become more prevalent among young disability pensioners. High risks of suicidal behaviour have remained over time among young adults with DP due to a range of psychiatric diagnoses. This underscores the emergent need for better support and treatment.
